# Black Soldier Fly (*Hermetia illucens*) Protein Concentrates as a Sustainable Source to Stabilize O/W Emulsions Produced by a Low-Energy High-Throughput Emulsification Technology

**DOI:** 10.3390/foods10051048

**Published:** 2021-05-11

**Authors:** Junjing Wang, Morane Jousse, Jitesh Jayakumar, Alejandro Fernández-Arteaga, Silvia de Lamo-Castellví, Montserrat Ferrando, Carme Güell

**Affiliations:** 1 Departament d’Enginyeria Química, Universitat Rovira i Virgili, 43007 Tarragona, Spain; junjing.wang@urv.cat (J.W.); jitesh.jayakumar@urv.cat (J.J.); silvia.delamo@urv.cat (S.d.L.-C.); montse.ferrando@urv.cat (M.F.); 2 AgroParisTech, 75005 Paris, France; morane.jousse@agroparistech.fr; 3 Departamento Ingeniería Química, Facultad Ciencias, Campus Universitario Fuentenueva, Universidad de Granada, 18071 Granada, Spain; jandro@ugr.es

**Keywords:** black soldier fly, insect protein, techno-functional properties, membrane emulsification, dynamic membrane

## Abstract

There is a pressing need to extend the knowledge on the properties of insect protein fractions to boost their use in the food industry. In this study several techno-functional properties of a black soldier fly (*Hermetia illucens*) protein concentrate (BSFPC) obtained by solubilization and precipitation at pH 4.0–4.3 were investigated and compared with whey protein isolate (WPI), a conventional dairy protein used to stabilize food emulsions. The extraction method applied resulted in a BSFPC with a protein content of 62.44% (Kp factor 5.36) that exhibited comparable or higher values of emulsifying activity and foamability than WPI for the same concentrations, hence, showing the potential for emulsion and foam stabilization. As for the emulsifying properties, the BSFPC (1% and 2%) showed the capacity to stabilize sunflower and lemon oil-in-water emulsions (20%, 30%, and 40% oil fraction) produced by dynamic membranes of tunable pore size (DMTS). It was proved that BSFPC stabilizes sunflower oil-in-water emulsions similarly to WPI, but with a slightly wider droplet size distribution. As for time stability of the sunflower oil emulsions at 25 °C, it was seen that droplet size distribution was maintained for 1% WPI and 2% BSFPC, while for 1% BSFPC there was a slight increase. For lemon oil emulsions, BSFPC showed better emulsifying performance than WPI, which required to be prepared with a pH 7 buffer for lemon oil fractions of 40%, to balance the decrease in the pH caused by the lemon oil water soluble components. The stability of the emulsions was improved when maintained under refrigeration (4 °C) for both BSFPC and WPI. The results of this work point out the feasibility of using BSFPC to stabilize O/W emulsions using a low energy system.

## 1. Introduction

The Food and Agriculture Organization (FAO) of the United Nations reports that the global population is likely to grow up to nine billion by 2050 [1,2] inducing a dramatic increase of food demand. There is a general agreement that the conventional protein sources will not be able to provide the approximately 260 million tons of proteins required by 2050, hence, there is a need to explore alternative protein sources that are both sustainable and possess high nutritional value. Beans and legumes containing high protein content (e.g., beans 23.5%, lentils 36.7%, and soybean 41.1% in dry matter) involved in the human diet since ancient times can be a good alternative, and soy is nowadays the most valuable protein source for feed; however, to rely solely on plant proteins to fill the expected protein gap puts huge pressure on the already pressured agricultural fields and raises serious environmental concerns [3,4,5,6,7,8].

Among the alternative protein sources, edible insects have drawn great attention in recent decades and FAO has claimed that edible insects are a good source of protein for dietary purposes [6]. Even though entomophagy, eating insects as foods, is being practiced by more than two billion people worldwide, most western developed countries have serious objections to consuming edible insects as a result of distaste and disgust about their nature and appearance [6,9,10,11,12,13]. Some studies showed that insect consumption was more acceptable in a masked way or when it was invisible in the food [11,14,15], which encouraged the integration of edible insects as milled powders and/or pastes in food products, as well as using their different functional fractions such as proteins, fatty acids, and chitin [16,17,18,19]. Moreover, in January 2021, the EFSA Panel on nutrition has adopted a scientific opinion on the safety of yellow mealworm as a novel food pursuant to Regulation (EU) 2015/2283.

There are several applications of insect functional fractions, from a recent study on the production of nano-emulsions from insect oils with potential application as drug/bioactive delivery systems [20] to other studies that explore the antimicrobial activity of peptides purified from insect proteins [21]. There are several studies testing protocols for extracting/purifying the protein fraction from crude insect meal for black soldier fly (*Hermetia illucens*), mealworm (*Tenebrio militor*), grasshopper (*Schistocerca gregaria*), honey bee (*Apis mellifera*), locust (*Locusta migratoria*), and cricket (*Gryllodes sigillatus*) [16,18,22,23,24,25]. Their procedures were based on the solubilization of protein in aqueous solution by adjusting pH values. Most of the studies on protein extraction/purification explore the potential functionality of the protein fractions. Gould and Wolf [18] used *T. molitor* protein as an emulsifier to stabilize sunflower oil-in-water emulsions produced by mechanical stirring; they obtained stable emulsions with droplet size smaller than emulsions stabilized with whey protein. Mishyna et al. [22] concluded that both *S. gregaria* and *A. mellifera* protein extracts have an emulsifying capacity comparable to whey protein; however, the insect source leads to some differences in the stability of the emulsions. Insect protein fractions have also been investigated with promising results as an ingredient in food formulations to replace meat in sausages [26] and meat batters [27]. Therefore, insect proteins, besides being used as a protein source, have the potential to be used in food and feed formulations to replace conventional proteins (such as dairy proteins) as gelling and emulsifying agents. This potential and the variations in the properties depending on the insect species and the development stage deserve more research studies to better understand their techno-functional properties, which will be highly relevant for food/feed industry regarding the preparation, processing, and storage of their edible insect food products.

Black soldier fly (*Hermetia illucens*) is a true fly belonging to the family Stratiomyidae, whose larvae contain 42% crude protein and 29% fat on average in the dry matter [28]. Although the protein content in black soldier fly (BSF) larvae is lower than in insects from the orthoptera species such as adult locusts, grasshoppers, and crickets which were reported to have up to 77% protein content in the dry matter [29], the advantage of BSF is the survival rate and the efficiency of converting organic materials into their own biomass [4,5]. Promising data on techno-functional properties of BSF protein fractions has been reported by Bußler et al. [16], while Caligliani et al. [17] presented a systematic approach for BSF meal fractionation, and suggested the use of enzymatic treatment to tailor the properties of the protein fractions. Even though the data regarding the emulsifying activity of BSF protein extracts is scarce, its potential to replace conventional dairy proteins, such as whey protein, in food/feed formulations is worthy to study.

As for the emulsification process, membrane emulsification is a widely used technique with advantages such as reduced mechanical stress, low energy consumption, and uniform droplet formation [30]. One of the reported drawbacks of membrane emulsification is the low productivity, which can prevent widespread application in the industry. Recently, dynamic membranes of tunable pore size (DMTS) have been applied to produce lemon oil emulsions [31] and double emulsions encapsulating carob polyphenols [32] in premix mode, that is, producing a coarse emulsion which is then refined by passing through the microstructured system. The DMTS system consists of a layer of glass microbeads supported by a nickel microsieve placed on the bottom of the membrane module. Because of this configuration, the DMTS system is easy to clean and re-use, and most importantly, requires low-energy input and yields high fluxes, showing its potential as a sustainable emulsification technology for industrial applications. This emulsification system has already been proved successful to produce sunflower oil-in-water emulsions stabilized with legume proteins [33], but it has never been tested for insect proteins.

This research aimed to assess the potential of black soldier protein extracts to stabilize O/W emulsions produced by a low-energy high-throughput emulsification technology. To achieve this goal, first, a protocol for protein extraction was implemented to obtain BSF protein concentrate (BSFPC), second, relevant techno-functional properties (solubility, foaming capacity and foam stability, emulsifying activity, water/oil binding capacity, and interfacial tension) of BSFPC were obtained and compared to whey protein isolate, and third, sunflower and lemon oil emulsions were produced by the DMTS system and compared with whey protein. This study provides relevant data towards the use of more sustainable protein sources and technologies in food production.

## 2. Materials and Methods

### 2.1. Materials

Partially defatted black soldier fly powder (BSF powder) was kindly provided by Hexafly (County Meath, Ireland). Sodium hydroxide pellets (NaOH, Chem-Lab NV, Zedelgem, Belgium) and hydrochloric acid (37–38% HCl, J.T. Baker, Griesheim, Germany) were used for BSF protein extraction. Hexane (≥99%, Sigma-Aldrich, Poznań, Poland) was used for removing lipid fraction. The BCA (bicinchoninic acid) assay kit (Pierce Biotechnology, Thermo Scientific, Rockford, IL, USA) was used for protein quantification giving the results in bovine serum albumin (BSA) equivalent value. Note that BSF concentrations that are BSA eq % are given hereinafter as % for simplicity. Phosphate buffer (pH 7) was prepared using sodium phosphate dibasic heptahydrate (HNa_2_O_4_P7H_2_O, ACROS, Barcelona, Spain) and sodium phosphate monobasic monohydrate (H_2_NaO_4_PH_2_O, ACROS, Barcelona, Spain). Acetic buffers (pH 3 and 5) were prepared with sodium acetate (Sigma-Aldrich, USA) and acetic acid (96%, Panreac, Barcelona, Spain). Buffers for pH 9 and 11 were prepared by sodium hydroxide (Chem-lab, Zedelgem, Belgium) and sodium tetraborate (ACROS, Spain). Whey protein isolate (WPI) was purchased from Davisco Foods International, Inc. (97.6%, Lot.JE151-4-420, Eden Prairie, MN, USA). Sunflower oil used as the oil phase for the O/W emulsions was purchased from a local supermarket (Borges S.A., Tarragona, Spain) and lemon oil (24L120 Limón Aroma) was ordered from Dallant S.A., Barcelona, Spain.

### 2.2. Protein Extraction

The protein extraction process was based on Zhao et al. [34] with some modifications. A total of 30 g of BSF powder was mixed with 150 mL of 0.25 M NaOH solution and the mixture was heated to 40 °C for one hour with constant agitation at 400 rpm on a magnetic stirrer (RCT ST, IKA, Staufen, Germany). The mixture was centrifuged (Meditronic 7000599, J.P. SELECTA, Barcelona, Spain) at 4490 rpm for 15 min and the lipid fraction on the top was carefully separated by pipets. The pellet was reserved for further extraction (twice more), while the pH of the supernatant was adjusted to 4.0–4.3 by adding 37% HCl and 1N HCl successively, followed by centrifugation (3750 rpm, 15 min) to obtain the precipitated proteins. After the centrifugation, the precipitated proteins were collected in plastic petri dishes and were kept at −60 °C until freeze-drying. The precipitated proteins were freeze-dried (LYOQUEST-85 PLUS, Telstar, Barcelona, Spain) for 24 h at 0.2 mbar with the plates heated to 20 °C. Freeze-dried samples were combined, ground, and defatted. Defatting was carried out by stirring 50 g of freeze-dried powder and 250 mL of hexane for one hour, after which powders were settled down and the hexane layer was decanted. This procedure was repeated until no color was observed in the hexane layer. The remaining hexane in the powders was evaporated in the fume hood for 2 days. Next, samples were collected and were kept in a desiccator at 4 °C until being used.

The yield of the extraction process and the yield of the protein extraction in this study were defined as Equations (1) and (2), respectively.

(1) $$\%\mathrm{Extraction}\;\mathrm{yield}=\frac{\mathrm{gram}\;\mathrm{of}\;\mathrm{BSFPC}\;\mathrm{output}}{\;\mathrm{gram}\;\mathrm{of}\;\mathrm{BSF}\;\mathrm{powder}\;\mathrm{input}}\times 100$$

(2) $$\%\mathrm{Protein}\;\mathrm{yield}\;=\frac{\mathrm{gram}\;\mathrm{of}\;\mathrm{protein}\;\mathrm{in}\;\mathrm{BSFPC}\;\mathrm{output}}{\;\mathrm{gram}\;\mathrm{of}\;\mathrm{protein}\;\mathrm{in}\;\mathrm{BSF}\;\mathrm{powder}\;\mathrm{input}}\times 100$$

### 2.3. Characterization of the BSF Powder and the BSFPC

#### 2.3.1. Amino Acid Composition, Total Nitrogen, and Protein Content

Total nitrogen and amino acid contents were analyzed by AGROLAB Ibérica S.L.U (Tarragona, Spain) based on Kjeldahl and the European Union Commission Regulation REG(UE) 152/2009, III, F: 2009-02 methods, respectively. Nitrogen to protein conversion factor, Kp, was calculated from the ratio of the sum of amino acid residue weights to nitrogen content [35]. The calculated Kp factor was used to estimate the protein content in the samples.

#### 2.3.2. Fourier Transform Mid Infrared Spectroscopy (FTIR)

The BSF powder and the BSFPC obtained after freeze-drying and defatting were analyzed by following the protocol of Mellado-Carretero et al. [36] to qualitatively determine the efficiency of the defatting process. For the acquisition of the spectral profiles, 4 mg of each sample was taken randomly and placed onto the sample stage of a portable spectrometer Cary 630 (Agilent Technologies Spain SL, Madrid, Spain), equipped with a single bounce ATR diamond crystal accessory and a deuterated triglycine sulfate (DTGS) detector. A pressure clamp was used to ensure optimal contact between samples and the diamond crystal. A background scan was extracted from every sample scan to prevent the effect of environmental changes. Spectra were acquired from 4000 to 800 cm^−1^ with 8 cm^−1^ of resolution using MicroLab PC software (Agilent Technologies SL, Madrid, Spain). 

### 2.4. Techno-Functional Properties of the BSFPC

#### 2.4.1. Protein Solubility

Protein solubility was examined with the similar method as described in the literature [22]. As it is pH, temperature, and ionic strength dependent, the measurements were performed at room temperature and different pH buffers with the same molarity. A sample of BSFPC (0.2 g of powder) was dispersed into 10 mL of 0.2 M buffer (pH 3, 5, 7, 9, and 11) and stirred for 2 h. The mixtures were centrifuged (Biocen 22R, Orto Alresa, Madrid, Spain) at 3250 g for 20 min. The protein content in the supernatants was quantified using BCA assay kit. All the experiments were performed at least in duplicate. The protein solubility was calculated as Equation (3).

(3) $$\%\mathrm{PS}=\frac{\mathrm{Soluble}\;\mathrm{protein}\;\mathrm{in}\;\mathrm{the}\;\mathrm{supernatant}}{\mathrm{Total}\;\mathrm{protein}\;\mathrm{content}\;\mathrm{in}\;\mathrm{the}\;\mathrm{powder}}\times 100$$

#### 2.4.2. Isoelectric Point (pI) Determination

pI of soluble protein fraction from BSFPC was determined by zeta potential using Zetasizer Nano-ZS (Malvern Instruments, Worcestershire, UK). A total of 0.1% of BSFPC soluble protein solution was prepared at pH 7, and the pH was subsequently adjusted to 5.5, 5.0, 4.5, 4.0, and 3.5 by adding 1 N HCl and 0.01 N HCl gradually. Zeta potential of supernatant after centrifugation at 3250 g for 20 min was measured and plotted against pH. The isoelectric point set to the pH region where the Zeta potential was close to 0.

#### 2.4.3. Water and Oil Binding Capacity

Water binding capacity (WBC) and oil binding capacity (OBC) were analyzed by the method as reported in the literature [25]. Briefly, for WBC, 0.5 g of powders were mixed with 2.5 mL of 0.2 M phosphate buffer (pH 7) and vortexed in a centrifugation tube for 60 s followed by centrifugation at 3250 g for 20 min at room temperature. The supernatant was decanted and the tube with the residual pellet was placed upside down on a filter paper for 60 min, to drain the residual non-bound water, before recording the weight. The WBC is calculated as shown in Equation (4),

(4) $$\mathrm{WBC}\;\left[\frac{g_{water}}{g_{DM}}\right]=\frac{m_{1}-m_{0}}{m_{DM}}\times 100$$

where $m_{0}$ is the initial weight of the sample, $m_{1}$ is the weight of residual after 60 min, and $m_{DM}$ is the dry matter of the initial sample which can be calculated by measuring the weight changes of the fresh sample being kept in the oven (UN55, Memmert, Büchenbach, Germany) at 105 °C until no difference in weight is observed.

As for OBC, similarly, 0.5 g powders were mixed with 2.5 mL of commercial sunflower oil and vortexed for 60 s twice with 5 min of pause in between. The rest of the procedure is identical to WBC analysis using Equation (5) for the calculation. Both WBC and OBC analyses were done in triplicate.

(5) $$\mathrm{OBC}\;\left[\frac{g_{oil}}{g_{DM}}\right]=\frac{m_{1}-m_{0}}{m_{DM}}\times 100$$

#### 2.4.4. Foaming Capacity (FC) and Foam Stability (FS)

The foaming properties were analyzed following the literature [22] with minor modifications. A sample of 20 mL of 0.1% BSFPC solution prepared with 0.2 M pH 7 buffer was placed in a 50 mL plastic tube and subjected to vigorous rotor-stator homogenization (Ultra Turrax T18 digital, IKA, Staufen, Germany) at 1200 rpm for 2 min. The height of the foam layer after 10 s and 120 min was recorded. FC and FS (from experiments run in duplicate) were calculated using Equations (6) and (7), respectively [22],

(6) $$\%\mathrm{FC}\;=\frac{H_{t}}{H_{0}}\times 100$$

(7) $$\%\mathrm{FS}=\frac{FC_{120}}{FC_{0}}\times 100$$

where $H_{0}$ is the initial height of protein solution in the tube, $H_{t}$ is the height of generated foam after agitation, $FC_{0}$ is the initial FC (after 10 s), and $FC_{120}$ is the one obtained after 120 min.

#### 2.4.5. Emulsifying Activity (EA)

EA was evaluated at different protein concentrations (0.1%, 0.5%, and 1.0%) of both BSFPC and WPI using the method described by Purschke et al. [25]. Briefly, in a beaker 10 mL of protein solution and 10 mL of sunflower oil were homogenized using ULTRA TURRAX at 11,000 rpm for 30 s. An aliquot of 10 mL of the emulsion was transferred into a 15 mL scaled tube and centrifuged at 3250 g for 20 min at room temperature. Triplicates were performed for each sample. The height of the emulsified layer was noted, and the EA was calculated using Equation (8) [25],

(8) $$\%\mathrm{EA}\;=\frac{H_{EL}}{H_{S}}\times 100$$

where $H_{EL}$ is the height of emulsified layer and $H_{S}$ is the total height of solution in the tube.

#### 2.4.6. Interfacial Tension Analysis

The pendant drop method was applied to monitor the interfacial tension changes of the interface of commercial sunflower oil and water phase by the tensiometer (CAM 200, KSV instrument, Espoo, Finland) coupled with the software CAM 2008. A capillary syringe filled up with water or protein solution (0.1% *w/w*) with a plastic cylindric tip (diameter 0.71 mm) was fitted in the tensiometer. The tip was immersed in the sunflower oil in a quartz cuvette. A pendant drop was generated by a capillary syringe with a controlled volume of 15 μL (±2 μL), and the interfacial tension was recorded immediately and every 5 min for 90 min or until it reached equilibrium. Reflective indices of 1.480 and 1.475 for sunflower oil and lemon oil were used for the calculation. Duplicates were performed for each water solution.

### 2.5. Premix Membrane Emulsification

#### 2.5.1. Preparation of O/W coarse emulsion

A total of 150 g of O/W emulsion was prepared based on the formulation in Table 1. The whey protein solution was prepared by dissolving the desired amount of WPI in deionized water (or in 0.2 M phosphate buffer pH 7) and stirring for 2 h. The solution was kept in the fridge overnight for complete hydration. BSFPC solution was prepared by dissolving BSFPC powder and stirring for 1 h followed by the pH adjustment to 7 by 1 M NaOH. After further stirring for 2 h, the solution was kept overnight in the fridge. Protein concentration was quantified using the BCA assay kit after centrifugation twice at 3750 rpm for 15 min, and the required concentration of BSFPC solution was obtained by dilution with deionized water (or 0.2 M phosphate buffer pH7). The coarse emulsion was prepared by homogenizing the oil phase and water phase in a beaker using a rotor-stator homogenizer (Ultra Turrax T18 digital, IKA, Staufen, Germany) at 15,800 rpm for 2 min and at 15,400 rpm for 1 min with 30 s of pause every 1 min to avoid temperature rising.

#### 2.5.2. Emulsification with Dynamic Membranes of Tunable Pore Size (DMTS)

**Figure 1:**
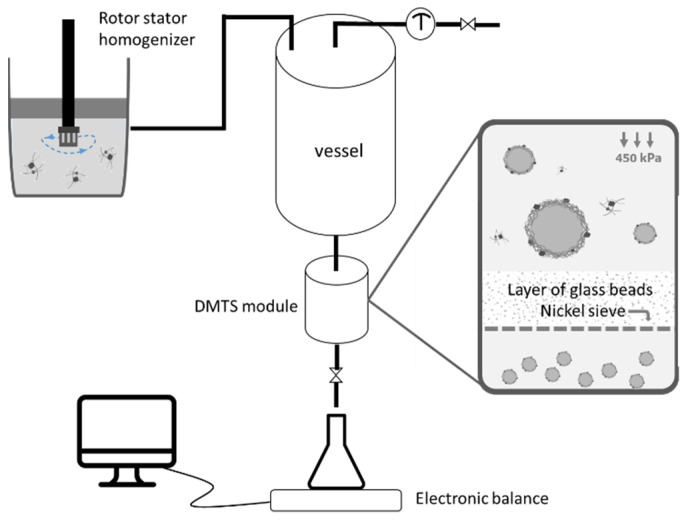
Premix emulsification and dynamic membrane of tunable pore size emulsification setup.

In a cylindric module, 2 g of 94.2 μm glass microbeads (resulting in a height of 8.3 mm layer) were placed on top of a nickel sieve having a pore size of 289 × 13 μm (length × width) with a thickness of 120 μm as illustrated on Figure 1. A pressure vessel was connected to the DMTS module where the coarse emulsions were pressurized by nitrogen to pass through the micron-sized interstitial voids formed by the glass microbeads layer to achieve droplet breakup. The emulsions were collected in an Erlenmeyer placed above an electronic balance to record the mass gain over time. The same procedure was repeated four times (five emulsification cycles in total) to further refine the emulsions. The interstitial void diameter ($d_{v}$) of the channels formed by glass microbeads was 78.4 μm, calculated as Equation (9),

(9) $$d_{v}=\frac{4\varepsilon }{\left(1-\varepsilon \right)6/d_{b}}$$

where $d_{b}$ is the microbeads diameter (94.2 μm) and *ε* is the porosity (0.55), which can be calculated using the particle ($\rho_{p}$) and bulk ($\rho_{b}$) densities of glass microbeads using Equation (10).

(10) $$\varepsilon =1-\frac{\rho_{b}}{\rho_{p}}$$

Transmembrane flux, $J_{DMTS}$, was calculated using Equation (11),

(11) $$J_{DMTS}=\frac{\phi }{\rho_{e}A}$$

where ϕ is the mass flow rate acquired from the mass/time data recorded with the electronic balance, $\rho_{e}$ is the emulsion density, A is the effective surface area of the DMTS.

The dynamic membrane was disassembled once the emulsification was completed (5 cycles) and the nickel sieve and glass microbeads were reused after cleaning based on the protocol applied by Kaade et al. [37]. Dishwashing detergent and ethanol were used to clean glass microbeads.

#### 2.5.3. Particle Size and Distribution

The particle size distribution of O/W emulsions was measured after every emulsification cycle by laser diffraction using Mastersizer 2000 (Malvern Instruments, Worcestershire, UK). Particle reflective indices were set to 1.480 and 1.475 for sunflower oil and lemon oil, respectively, and the dispersant reflective index was set to 1.330. Mean droplet size and droplet size dispersion can be calculated, and expressed as Sauter mean diameter *d*_3,2_ (Equation (12)) and the span factor (Equation (13)), respectively,

(12) $$d_{3,2}=\frac{6}{S_{v}}={\left(\overset{n_{s}}{\underset{i=1}{\sum }}\frac{v_{i}}{d_{i}}\right)}^{-1}$$

where $S_{v}$ is the droplet surface area per unit volume, $v_{i}$ is the volume fraction of droplets in the *i*th size class of the discretized distribution, $d_{i}$ is the mean droplet diameter in that class and $n_{s}$ is the number of size classes,

(13) $$\delta =\frac{d_{90}-d_{10}}{d_{50}}$$

where $d_{x}$ is the droplet diameter corresponding to x% volume on a cumulative droplet size distribution curve.

#### 2.5.4. Zeta Potential

Zeta potential of emulsions was measured using Zetasizer Nano-ZS (Malvern Instruments, Worcestershire, UK). Samples were diluted 100 times by deionized water. The same values of reflective indices for the particle (sunflower or lemon oil droplets) and dispersant as those used in Section 2.5.3 were also applied here.

#### 2.5.5. Analysis of Emulsion Stability

Several 10 mL aliquots were collected in tubes for every freshly produced emulsion. Emulsions were kept at room temperature (25 °C) and in refrigerator (4 °C) for 7 days. Droplet size distribution, as well as zeta potential were measured after 1, 3, and 7 days.

### 2.6. Statistical Analysis

The data described are mean ± standard deviation. Significant differences between the groups were determined using ANOVA and Tukey test (*p* < 0.05).

## 3. Results and Discussion

### 3.1. Chemical Composition of BSF Powder and BSFPC

#### 3.1.1. Amino Acids, Total Nitrogen, and Protein Content

The protein content in the BSFPC was determined based on the amino acids and total nitrogen contents and compared with the BSF powder. Nitrogen-to-protein conversion factor (Kp) for insects is lower than the conventional value (Kp = 6.25) due to the existence of non-protein nitrogen compounds such as chitin [38]. Therefore, it is necessary to recalculate the Kp from amino acids contents as described in Section 2.3.1. The Kp values calculated shown in Table 2 are slightly lower than the true values as they were calculated based on only 17 amino acids, which led to slightly lower amount of protein contents estimated. Most of the amino acid contents measured in BSFPC were higher than those in the BSF powder resulting in an increase of the Kp value from 4.71 to 5.36. It indicates that the extraction process effectively reduced non-protein nitrogen compounds [17]. Based on the calculated Kp values and the total nitrogen contents of the BSF powder and BSFPC, the protein contents were 39.00% and 62.44%, respectively, which are comparable to those (36.7% and 67.6%) reported by Janssen et al. [38] using pH 6 phosphate buffer in protein extraction. 

Results showed the essential amino acid contents in BSFPC meet the FAO recommendation of protein quality. Some amino acids were two (histidine and threonine) and three times (phenylalanine and tyrosine) higher than the recommended values, except for undetermined content of tryptophan (Table 2). It also showed comparable protein quality to soybean, a plant sourced agriproduct. However, BSFPC contained slightly lower amount of leucine and lysine compared to casein protein. The protein extraction process resulted in a loss of total amount of sulphur amino acids (methionine and cysteine), which decreased from 35.4 mg/g protein in BSF powder to 28.2 mg/g protein in BSFPC, which is mainly due to the reduction of cysteine (from 0.43% DM in BSF powder to 0.36% DM in BSFPC). Even though cysteine is not classified as an essential amino acid, it is the key amino acid conforming disulphide bonds in high order protein structure [39]. The reduction of cysteine can be attributed to the strengthened protein’s tertiary and quaternary structure with multiple disulphide bridges of cysteines, which makes it difficult to extract out [40].

In this study, a 35.8% of extraction yield and a 59.3% of protein yield were obtained which was higher than the protein yield reported by Janssen et al. [38] (17.1%) and in the range (47% to 91%) of which obtained by Caligiani et al. [17] using three different extraction methods. As for the studies on other insect species, 17% to 30.8% of extraction yields and 26.4% to 59.9% of protein yields were reported from *T. molitor* [16,18,41,42], *Z. morio*, *A. diaperinus* [43], *A. domesticus*, *B. dubia* [42], *L. migratoria* [23], *S. gregaria*, and *A. mellifera* [22].

#### 3.1.2. Attenuated total Reflectance Fourier Transform Mid-Infrared Spectroscopy (ATR-FT-MIR)

**Figure 2:**
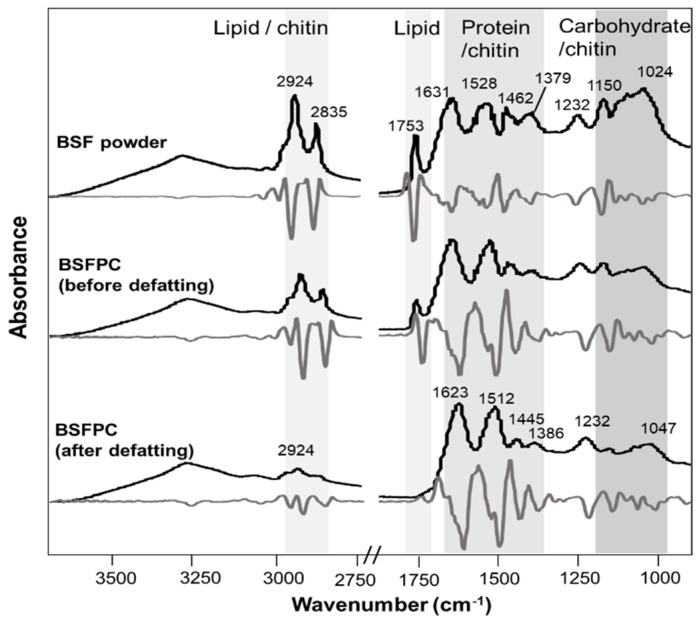
Attenuated total reflectance Fourier transform mid infrared spectroscopy (FTIR) raw spectra (black line) of BSF (black soldier fly) powder and BSFPC (black soldier fly protein concentrate) before and after defatting and the corresponding Savitzky–Golay’s second derivatives (grey line).

Figure 2 shows the attenuated total reflectance FTIR raw spectra and Savitzky–Golay’s second derivatives (11 points) of BSF powder and BSFPC before and after defatting. Raw spectra and second derivatives of BSF powder and BSFPC before and after defatting show four important spectral regions: 2930–2850 cm^−1^, 1753 cm^−1^, 1630–1510 cm^−1^, and 1150–1020 cm^−1^. These spectral regions have also been reported by other authors that have analyzed different insect species, *P. succincta*, *C. roseapbrunner* [45], *T. molitor*, *A. diaperinus*, *G. sigillatus*, *A. domesticus*, and *L. migratoria* [36]. Strong IR bands at 2924 cm^−1^, 2853 cm^−1^, and 1753 cm^−1^ linked to CH_2_ asymmetric stretching, CH_2_ symmetric stretching, and C=O stretching of lipids, respectively [46], are present in BSF powder and BSFPC before defatting spectra. In the spectrum of BSFPC after defatting, the IR band at 1753 cm^−1^ disappeared and the absorbance of the IR bands at 2924 cm^−1^, 2853 cm^−1^ significantly decreased. 

### 3.2. Techno-Functional Properties

**Figure 3:**
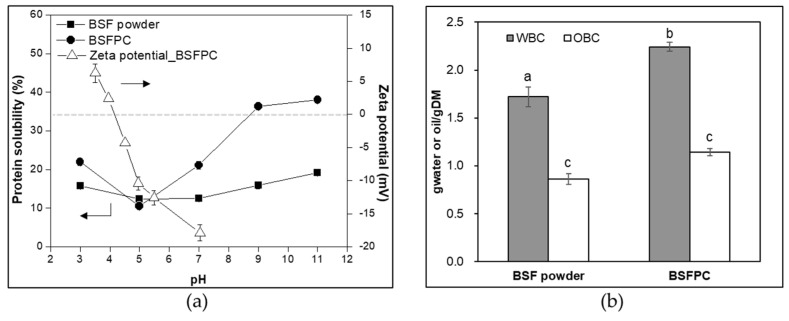
(**a**) Protein solubility at pH ranging from 3 to 11 and zeta potential at pH ranging from 3.5 to 7; (**b**) WBC (water binding capacity) and OBC (oil binding capacity) of BSF (black soldier fly) powder and BSFPC (black soldier fly protein concentrate). Different lowercase letters indicate significant differences at the *p* < 0.05 level.

**Figure 4:**
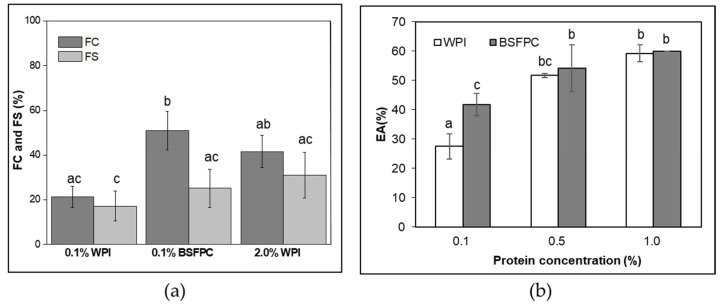
(**a**) FC (foaming capacity) and FS (foam stability) among different concentration of WPI (whey protein isolate) and BSFPC (black soldier fly protein concentrate); (**b**) EA (emulsifying activity) compared WPI with BSFPC. Different lowercase letters indicate significant differences at the *p* < 0.05 level.

To determine the techno-functional properties of BSFPC and assess its potential applications, solubility, WBC and OBC, FC and FS, EA and interfacial tension were analyzed and summarized in Figure 3 and Figure 4.

#### 3.2.1. Protein Solubility and Isoelectric Point (pI)

As for the protein application in aqueous media, solubility is of importance. Figure 3a shows that solubility of BSF powder and BSFPC depends largely on pH value, with the highest solubilities (19.1% and 38.0%) at pH 11, and lowest solubility (10.5% and 12.4%) at pH 5. Isoelectric point (pI) of BSFPC was determined to be in the range of pH 4.0 to 4.5 (Figure 3a), which explained the lowest solubility observed at pH 5. This is in agreement with the range of protein pI (4–5) found in the literature of *T. molitor* [16,24,47], *S. gregaria* [22,24], *A. domesticus* [24], *A. mellifera* [22], and *H. illucens* [16]. Overall, the solubility values for BSF powder and BSFPC are comparatively lower than the ones reported for *T. molitor* [16,47], and *S. gregaria* and *A. mellifera* [22]. Nevertheless, the BSFPC presented an improved protein solubility compared to the BSF powder shown by the two-fold increase of solubility at pH 7–11. Purschke et al. [23] also observed the synergic effect of ionic strength (1% NaCl to 3% NaCl) between pH 4 and pH 9 on protein solubility for *T. molitor* protein concentrate. Moreover, protein solubility can be improved after enzymatic hydrolysis of *A. domesticus* and *L. migratoria* powders [19,25]. None of these strategies were used in this study to increase the solubility of the BSFPC but they could be applied when solubility must be enhanced. Since some techno-functional properties such as foaming and emulsifying depend on the soluble protein fraction, based on the solubility results obtained, protein solutions at pH 7 were prepared to conduct foaming and emulsifying tests in the later sections.

#### 3.2.2. WBC and OBC

WBC and OBC are critical features of food ingredients in food processing and applications. They are related to the ability of taking up and retaining water and oil, respectively, which directly affect the texture and the flavor of the products, especially in meat and bakery [47]. As for foods with high protein content, protein molecule structure, amino acid composition, pH, hydrophilicity, and hydrophobicity on protein surface are determining factors of WBC and OBC [24,47]. BSFPC had WBC of 2.2 g water/g DM that is higher than the reported 0.4 g water/g DM and 1.5 g water/g DM for *T. molitor* protein extract and enzymatic hydrolysate of *L. migratoria* protein [16,23], respectively, but lower than hexane defatted *T. molitor* powders (2.7 g water/g DM) [48] (Figure 3b). As for the OBC, the value obtained for BSFPC (1.1 g oil/g DM) was higher than the reported one for *T. molitor* protein extract [16], in the range of the ones for defatted *T. molitor* [48], and lower than the one reported from enzymatic hydrolysate of *L. migratoria* protein [23]. The relatively higher WBC might be due to the higher protein content in the BSFPC which contains more hydrophilic groups to bind with water molecules. On the contrary, OBC can relate to the hydrophobic groups on the surface of protein molecules to bind with oil. Zielińska et al. [24] reported higher values of WBCs (2.18–3.95 g_water_/gDM) and OBCs (1.98–3.33 g_oil_/gDM) of protein extracts from *T. molitor*, *L. migratoria*, and *A. domesticus* than the ones found for BSFPC. The WBC and OBC of the BFS powder and BSFPC are comparable to plant-based flours such as wheat and rice, which were reported to have WBC from 1.4 to 1.9 g_water_/gDM, and OBC from 1.5 to 1.9 g_oil_/gDM, respectively [49]. Therefore, the BSFPC obtained in this study seems to be suitable in broad food applications entailing high protein content. 

#### 3.2.3. FC and FS

A total of 0.1 wt.% and 2 wt.% whey protein isolate (WPI) solutions and 0.1 wt.% soluble BSFPC solutions were compared in terms of FC and FS. As it is shown in Figure 4a, 0.1% BSFPC displayed higher FC (51%) than 0.1% WPI (21.4 %) and even than 2% WPI (41.7%). The mechanical agitation by Ultra Turrax introduced not only air bubbles but also energy into the whole system which can unfold the protein structure and change the protein conformation favoring the stabilization of air bubbles at the air-water interface. It is reported that protein extraction and enzymatic hydrolysis resulted in the generation of small peptides with surface-stabilizing residues which can rapidly diffuse onto the interface and rearrange the structure to improve FC [19,22,23,24]. Yi et al. [42] and Purschke et al. [25] discussed the impact of pH and ionic strength on foaming and found improved foaming behavior both at near protein pI and at increased NaCl concentration. Apart from the protein structure, the positive effect of carbohydrates on foaming was explained by Zielińska et al. [24] which might be also the case in this study due to the BSFPC contained 62.44% of protein and the remaining parts are possibly carbohydrates and soluble fibers. 

Regardless of the FC, 2% WPI solution showed the highest FS at 120 min after foam generation (31.1%), followed by 0.1% BSFPC (25.2%) and 0.1% WPI (17.3%). Due to the higher concentration of protein in the 2% WPI solution, the viscoelastic film formed at the air-water interface [22,25] was more durable. Nevertheless, BSFPC is considered to be suitable for preparing food products based on foams.

#### 3.2.4. EA

EAs of WPI and BSFPC at concentrations of 0.1%, 0.5%, and 1.0% were assessed as described at Section 2.4.5. BSFPC at 0.1% presented higher EA than 0.1% WPI; however, both were not able to emulsify all oil. A visible top oil layer was observed after centrifugation, which was more significant for 0.1% WPI. When the protein concentration was raised to 0.5%, the gap in EA between WPI and BSFPC was reduced (51.7% and 54.2%, respectively) and the EA values were nearly equal (59.2% and 60.0%) at 1.0% protein concentration. It is worth mentioning that 0.5% BSFPC was able to emulsify all oil fraction, whereas 0.5% WPI was not competent to do so until further increased to 1%.

The results of EAs showed the same trend as the FC of WPI and BSFPC discussed in the previous section. As both properties are dependent on the functionality of hydrophobic groups on the protein surface and its molecular flexibility, it can be assumed that BSFPC can be used in emulsification as other conventional emulsifiers, such as WPI. This was also explained by Zielińska et al. [24] who reported improved EAs in insect protein extracts compared to their whole fraction powders, which was due to the increased protein contents in the protein extracts, especially with the amount of the hydrophobic amino acids. Purscke et al. [25] studied the effect of pH and ionic strength on the EA of *L. migratoria* protein concentrates concluding EA increased with the increase of ionic strength, and the highest EA was reached near the isoelectric point of the protein. However, regarding the functionality of a protein to be used as an emulsifier, there are more factors to be considered, such as the stability of emulsions, droplet size distributions, and zeta-potential, which are explained in the later sections.

#### 3.2.5. Interfacial Tension

**Figure 5:**
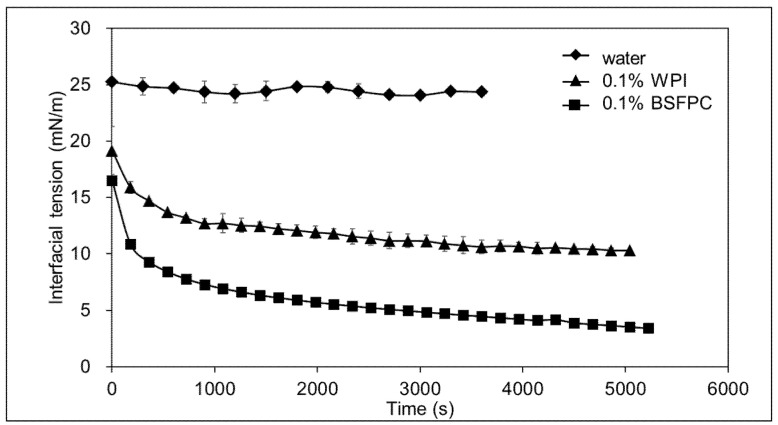
Interfacial tension between aqueous solutions of water, 0.1% WPI (whey protein isolate) and 0.1% BSFPC (black soldier fly protein concentrate), respectively, and sunflower oil (25 °C).

The effect of WPI and BSFPC on sunflower oil-water interfacial tension was analyzed as described in Section 2.4.6. As indicated in Figure 5, the interfacial tension of sunflower oil-water was around 25 mN/m and no significant reduction was observed due to the absence of surface-active compounds. The addition of WPI or BSFPC lowered the interfacial tension almost instantaneously to 13.7 and 8.4 mN/m, respectively. During the next 10 min, the interfacial tension decreased quickly, and from that point on the decrease was slower, reaching a plateau value of 10.3 mN/m and 3.4 mN/m for WPI and BSFPC, respectively. The dynamics of the interfacial tension is limited by the diffusion rate of emulsifiers and, subsequently, the time required to reach and adsorb at the interface. When analyzing the progress of interfacial tension in similar oil-water systems stabilized with food grade proteins [33] three different phases have been identified: (i) a lag-time controlled by the initial migration of protein molecules to the interface, (ii) a sharp decrease by absorption of protein molecules on interface, and (iii) a slow decrease to reach pseudo-equilibrium interfacial tension by rearrangement of protein molecules and multilayer-film formation. Results in Figure 5 show a two-phase process with no significant lag time, and a faster diffusion and adsorption of BSFPC resulted in lower interfacial values at time 0 than WPI. Differences in the molecular weight, protein composition, and structure may lead to differences in the performance of BSFPC to reduce the interfacial tension in the sunflower oil-water [18,50].

This finding agrees with the results reported by Gould and Wolf [18] who compared the effects of whey protein and *T. molitor* protein on the purified sunflower oil-water interface, and obtained equilibrium interfacial tensions of 13 mN/m and 12 mN/m for whey protein and *T. molitor* protein extract, respectively.

### 3.3. Dynamic Membrane of Tunable Pore Size (DMTS) Emulsification

After proving the emulsifying capacity of BSFPC at certain conditions of rotor-stator homogenization (see Section 3.2.4), we assessed their ability to stabilize emulsions produced with a low-energy membrane emulsification technique. Premix membrane emulsification with DMTS was used to obtain BSFPC and WPI-stabilized O/W emulsions formulated with sunflower (SO) or lemon oil (LO).

#### 3.3.1. Droplet Size Distribution

Both oils are widely used in the food industry in the form of an emulsion, although they show important differences in composition and water solubility. SO, broadly used as a medium for delivering lipophilic micronutrients such as carotenoids and vitamin E [51], is a complex mixture of fatty acids, totally immiscible in water, while LO, used as a flavoring and antimicrobial agent, is rich in terpenes and partially water soluble [52,53]. 

**Figure 6:**
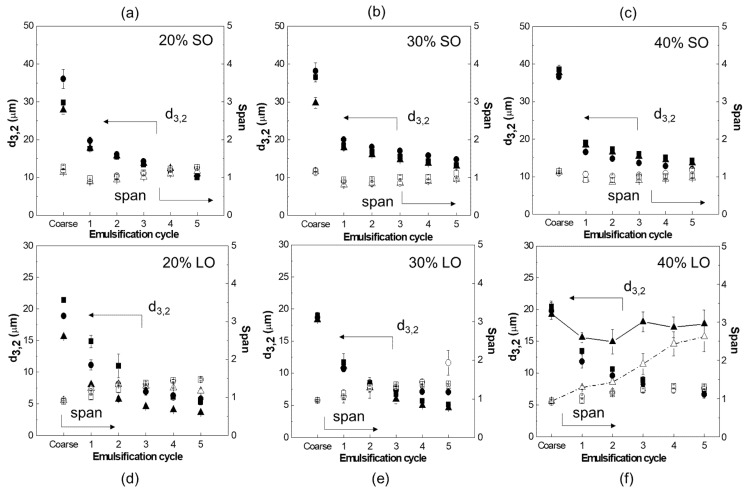
Sauter mean diameter (d_3,2_, full symbols) and span (empty symbols) versus the number of emulsification cycles for emulsions at different oils and oil fractions: (**a**) 20% SO (sunflower oil); (**b**) 30% SO; (**c**) 40% SO; (**d**) 20% LO (lemon oil); (**e**) 30% LO; and (**f**) 40% LO. (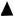 d_3,2_-1% WPI (whey protein isolate); 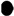 d_3,2_-1% BSFPC (black soldier fly protein concentrate); 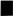 d_3,2_-2% BSFPC; 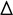 span-1%WPI; 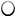 span-1% BSFPC; 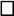 span-2% BSFPC).

As can be seen in Figure 6, BSFPC was able to stabilize O/W emulsions to a similar or even to a higher extent than WPI all along the emulsification process. Notice that all proteins were prepared with no buffering solution. In addition, the different nature of each oil strongly impacted the progress of droplet size and span, especially at the highest oil fraction. During DMTS emulsification, droplets break up as they go through the interstitial voids between the microbeads that make up the dynamic membrane, in this case with a mean diameter of 78.4 μm. In most cases, droplet size reduction followed an analogous pattern: a large droplet size reduction took place at the first emulsification cycle, after which there were only minor reductions with a slight increase in span, which agrees with several studies using the same set-ups [32,54,55].

As for SO emulsions, d_3,2_ and span reached values of 12.5 ± 1.8 μm and 1.1 ± 0.1, respectively, after five cycles of DMTS emulsification regardless of the emulsifier and oil fraction, even though d_3,2_ of the coarse emulsion (cycle 0) ranged from 28 μm to 40 μm for emulsions with 20% and 40% oil fraction, respectively. Therefore, the surface-active properties of BSFPC and WPI were able to stabilize even the highest oil-in-water interface area created during emulsification of 40% oil fraction emulsions.

Compared to SO, LO emulsions showed smaller droplet size of the coarse emulsions with values ranging from 18 μm to 22 μm because of the lower interfacial tension of the LO-water system (12.9 ± 0.2 mN/m [56] and 11.82 mN/m [57]) compared to the SO-water system (25 mN/m, Figure 5). After the refining process with DMTS, d_3,2_ decreased to values between 4 μm and 7 μm and span ranged from 1 to 2 when oil fraction was kept below 30% (Figure 6d–f). However, when the oil fraction increased to 40%, WPI stabilized emulsions could not further get refined in DMTS after three cycles what resulted in higher values of d_3,2_ and span than those obtained with BSFPC stabilized emulsions, which could be successfully refined over five emulsification cycles (Figure 6f). The poor performance of WPI as an emulsifier in the LO/W system could be linked to the chemical composition of LO, containing anhydrous acids and phenolic acids from the peel [58,59], able to diffuse in the water phase and reduce pH below the WPI isoelectric point, in a range of 4.8–5.1. The water phase of LO/W emulsions containing 40% oil fraction showed a pH of 5.03 that may cause aggregation and precipitation of whey proteins and, in turn, a reduction of their surface-active capacity. At these conditions, the slightly lower isoelectric point of BSFPC (pH 4.0–4.5) was more favorable to stabilize LO/W emulsions with a 40% oil fraction. 

**Figure 7:**
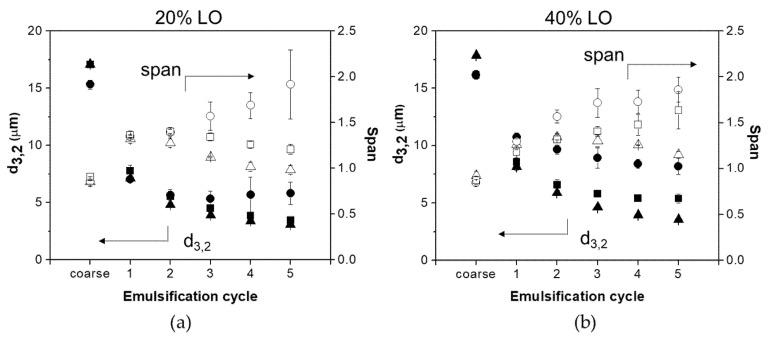
Sauter mean diameter (d_3,2_, full symbols) and span (empty symbols) versus the number of emulsification cycles for produced LO (lemon oil) emulsions with 0.2 M phosphate buffer in the water phase at different oil fractions: (**a**) 20% LO and (**b**) 40% LO. (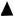 d_3,2_-1 % WPI (whey protein isolate); 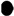 d_3,2_-1 % BSFPC (black soldier fly protein concentrate); 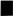 d_3,2_-2 % BSFPC; 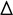 span-1%WPI; 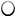 span-1% BSFPC; 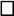 span-2% BSFPC).

To confirm the impact of pH on protein performance to stabilize LO/W emulsions, a set of experiments was carried out using 0.2 M phosphate buffer pH 7 as water phase. LO/W emulsions having 20% and 40% oil fraction were formulated with 1% WPI, 1% BSFPC, or 2% BSFPC. Figure 7 shows how, under these pH conditions, whey proteins were able to successfully stabilize LO/W emulsions during emulsification. After five emulsification cycles, emulsions with 20% oil fraction showed d_3,2_ of 3.1 μm and span of 0.98, while those emulsions with 40% oil fraction had a d_3,2_ of 3.6 μm and span of 1.15. Regarding BSFPC, although it also stabilized LO/W emulsions in the five cycles of DMTS emulsification, it was not able to maintain the span that increased over the process. The results obtained from WPI emulsified LO emulsions with 20% and 40% oil fractions were similar to what reported by Kaade et al. [31] which had slightly smaller d_3,2_ due to the smaller-size emulsifier Tween 20 was used.

#### 3.3.2. Productivity

**Figure 8:**
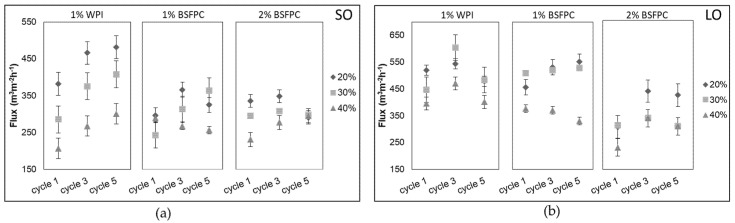
Transmembrane fluxes obtained at cycle 1, 3, and 5 during DMTS (dynamic membrane of tunable pore size) emulsifications of (**a**) SO (sunflower oil) emulsions and (**b**) LO (lemon oil) emulsions with different formulations.

The productivity of the emulsification process, measured as flux during emulsification, is a key parameter for process scale-up. Fluxes obtained during the fifth emulsification cycle of SO emulsions ranged from 206 m^3^m^−2^h^−1^ to 481 m^3^m^−2^h^−1^ and the ones of LO emulsions were between 231 m^3^m^−2^h^−1^ and 617 m^3^m^−2^h^−1^, depending on the oil fraction (Figure 8). The higher values obtained for LO emulsions can be attributed to the lower viscosity of this oil (1.41 mPa·s at 25 °C) [56] compared to the one of SO (48.8 mPa·s at 26 °C) [60] since as in any membrane system, flux is inversely proportional to the viscosity. Consequently, the lowest flux values always correspond to the emulsions with the highest oil fraction. As for the effect of the protein type and concentration, Figure 8 shows that regardless of the emulsion formulation, refining emulsions stabilized with WPI resulted in higher fluxes than the ones stabilized with BSFPC. Given that BSFPC had a protein content of 62.4% and the impurities can be carbohydrates and soluble fibers, it is thought that they contributed to increase the viscosity of the continuous phase. The effect of the viscosity of the continuous phase on the flux for the DMTS system has been previously seen by Kaade et al. [31], who reported higher fluxes during the emulsification of LO emulsions stabilized with 2 wt.% Tween 20 than the ones obtained using 1% WPI. Moreover, proteins and other compounds in BSFPC stabilized emulsions can also result in fouling of the DMTS system, compared to emulsions stabilized with WPI as already observed during premix emulsification with several microstructured systems [31,61,62,63].

#### 3.3.3. Stability of the Emulsions

**Figure 9:**
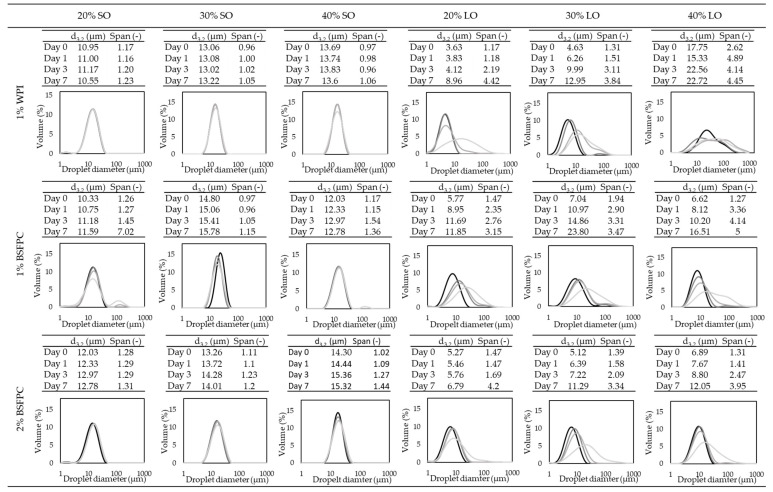
Particle size distribution of SO (sunflower oil) and LO (lemon oil) emulsions (no buffer added) stabilized by 1%WPI (whey protein isolate), 1% BSFPC (black soldier fly protein concentrate), and 2% BSFPC at day 0, day 1, day 3, and day 7 of storage at room temperature (25 °C). The color transparency scale indicates the length of storage time: from darkest to lightest refer fresh emulsion (day 0), day 1, day 3, and day 7.

**Figure 10:**
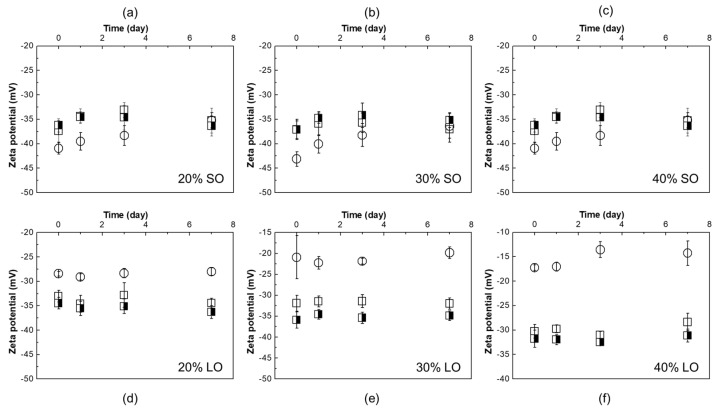
Zeta potential versus storage time at room temperature (25 °C) for produced emulsions (no buffer added) at different oils and oil fractions: (**a**) 20% SO (sunflower oil); (**b**) 30% SO; (**c**) 40% SO; (**d**) 20% LO (lemon oil); (**e**) 30 % LO; and (**f**) 40 % LO. (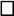 1% WPI (whey protein isolate); 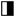 1% BSFPC (black soldier fly protein concentrate); and 2% BSFPC).

SO emulsions stabilized with WPI (1%) and BSFPC (1 and 2%) with oil fractions of 20%, 30%, and 40% were kept at room temperature (25 °C) for seven days. Samples were measured on days 1, 3, and 7 of storage to follow changes in the droplet size distribution. Although all the emulsions had creaming after one day of storage, it can be seen from the droplet size distribution measurements (Figure 9) that SO emulsions, in general, can maintain its d_3,2_ and span during seven days of storage at 25 °C with a minor increase in d_3,2_ (<2 μm) and span (<0.5). There was a moderate increase in span for the emulsions stabilized with 1% BSFPC with 20% SO at day 7 which can be the aggregation of oil droplets due to the protein-protein interaction [64]. In agreement with droplet size distribution evolution in time, zeta potential of 1% BSFPC SO emulsions kept at 25 °C was below −30 mV (Figure 10), the reference value for sufficient electrostatic repulsion to prevent droplet coalescence. SO emulsions stabilized by WPI showed a stronger surface repulsive effect (zeta potentials between −45 mV and −40 mV) than the ones stabilized by BSFPC (−40 mV to −35 mV).

**Figure 11:**
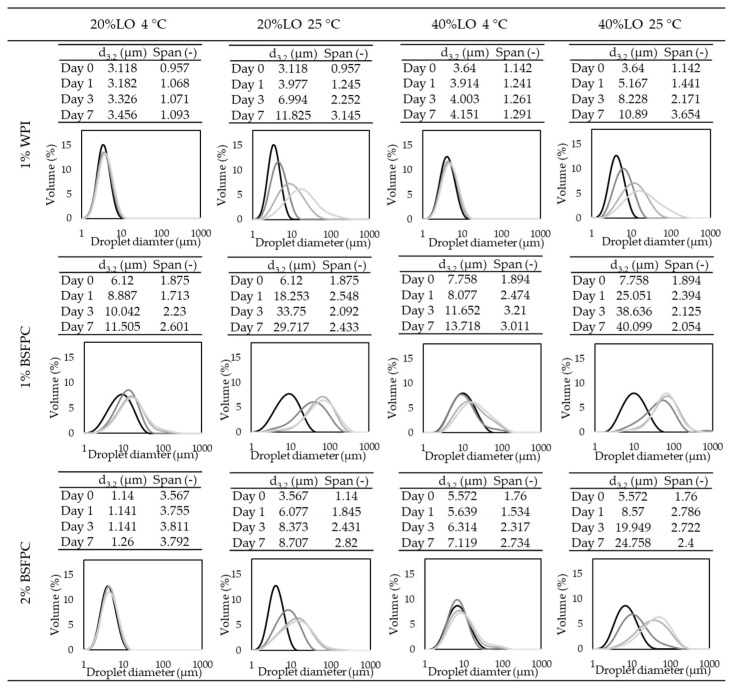
Particle size distribution of LO (lemon oil) emulsions stabilized by buffered 1% WPI (whey protein isolate), buffered 1% BSFPC (black soldier fly protein concentrate), and buffered 2% BSFPC after day 0 fresh emulsion, day 1, day 3, and day 7 of storage at room temperature (25 °C) and in fridge (4 °C). The color transparency scale indicates the length of storage time: from darkest to lightest refer fresh emulsion (day 0), day 1, day 3, and day 7.

As for LO emulsions, the ones prepared with unbuffered proteins were kept at room temperature (Figure 9), while the emulsions prepared with buffered proteins were kept at both room temperature and refrigeration (Figure 11). The emulsions without buffer showed a significant increase in d_3,2_ and span after seven days of storage at 25 °C, regardless of the protein used. These changes agree with the zeta potential values (−28.4 mV to −17.3 mV, Figure 10d–f) of WPI stabilized emulsions, which indicate a tendency to droplet coalescence. In LO emulsions the pH decreases to values close to the Ip of WPI, affecting the protein conformation and hence emulsion stability. Since the Ip of BSFPC used in this study is slightly lower than the one of WPI, the decrease of pH has a lower impact on the protein conformation. The emulsions showed a lower increase of d_3,2_ and span after seven days of storage at 25 °C than emulsions stabilized with WPI. For BSFPC the zeta potential values were maintained in the range of −35.9 mV to −30.3 mV for all the emulsions. As for the LO emulsions prepared with phosphate buffer, the ones with WPI showed a slight increase of d_3,2_ and span after seven days of storage at 25 °C and almost no changes when stored at 4 °C. The stability of these emulsions correlates well with their zeta potential values, −70 mV to −50 mV (Figure S1). However, when using a phosphate buffer to produce emulsions with BSFPC, d_3,2_ and span increased more than for the unbuffered emulsions at 25 °C. Decreasing the storage temperature had a positive effect on the emulsion stability, mainly for the lowest oil fraction. From these results, it seems that the BSFPC obtained in this work could be a better option for encapsulating lemon oil without the need of buffering the pH of the continuous phase. 

## 4. Conclusions

This study presents a holistic approach to the use of black soldier fly protein to stabilize food emulsions. The extraction process enabled enrichment of the protein content from 39% (original powder) to almost 63% thereby resulting in a protein concentrate (BSFPC). The essential amino acid profile of BSFPC is similar to soybean and casein and meets the FAO recommendation. As for the techno-functional properties of the BSFPC compared to WPI, it has been proven that BSFPC has higher FC and FS than WPI at low protein concentration (0.1%). Moreover, it was found that BSFPC has higher values of EA for low protein concentration (0.1%) than WPI and comparable values to WPI for 2% concentration. These results show the potential of BSFPC to be used in food formulations to replace totally or partially WPI. Moreover, the ability of BSFCP to lower the interfacial tension in the sunflower oil/water system as well as the values for WBC and OBC, point out the high potential of this protein to stabilize food emulsions. This has been proved using BSFCPC to stabilize emulsions with two oils frequently used in the food industry, such as sunflower oil and lemon oil. The emulsions have been produced using a low-energy high-throughput system previously tested with conventional food emulsifiers. Droplet size distribution and fluxes obtained for sunflower oil emulsions stabilized with BSFPC are comparable to the ones obtained with emulsions stabilized with WPI. For lemon oil emulsions, however, BSFPC successfully reduced droplet size distribution of emulsions with 20% to 40% oil fraction, while the ones produced with WPI for 30% and 40% oil fraction clearly showed an increase in the droplet size distribution after each emulsification cycle. It has been seen that since lemon oil is partially soluble in water when the emulsions have more than 30% oil fraction, there is a decrease in the pH of the emulsion. For WPI this pH decrease leads to a value close to pI of the protein, and therefore lowering its ability to stabilize the oil-water interface. Since the BSFPC has a lower pI, this phenomenon is not that important. As for the storage stability of the emulsions, the results point out that BSFPC has comparable results to WPI for sunflower oil at 4 °C. For lemon oil, or at higher temperatures (25 °C) WPI can better maintain the droplet distribution if the pH can be controlled. Even though further research is required to improve the protein extraction process to improve its solubility, the results of this study show the potential of BSFPC to become a sustainable protein for the food industry.

## Figures and Tables

**Table 1:** Composition of O/W emulsions.

Oil Phase	Oil Fraction	Emulsifier in Water Phase	Water Phase Medium
Sunflower oil or lemon oil	20 wt.%, 30 wt.%, or 40 wt.%	1% WPI	Deionized water or 0.2 M phosphate buffer pH7 ^1^
		1% BSFPC	
		2% BSFPC	

^1^ 0.2M phosphate buffer pH 7 was tested only for 20 and 40 wt.% lemon oil emulsions stabilized by 1% WPI, 1% BSFPC and 2%BSFPC.

**Table 2:** Amino acid composition, total nitrogen, and calculated Kp (nitrogen to protein conversion factor) value and protein content for BSF (black soldier fly) powder and BSFPC (black soldier fly protein concentrate), compared to soybean, casein, and FAO (Food and Agriculture Organization) recommendation for adults.

	BSF Powder (% DM)	BSFPC (% DM)	BSF Powder (mg/g Protein)	BSFPC (mg/g Protein)	2013 FAO (mg/g Protein)	Soybean (mg/g Protein) ^5^	Casein (mg/g Protein) ^5^
Essential amino acid							
His	1.71	2.60	43.8	41.6	16	25	32
Ile	2.12	3.68	54.4	58.9	30	47	54
Leu	3.36	5.80	86.9	92.9	61	85	95
Lys	3.06	5.12	78.5	82.0	48	63	85
Met+Cys ^3^	1.38	1.76	35.4	28.2	23	24	35
Phe+Tyr ^4^	5.23	9.43	134.1	151.1	41	97	111
Thr	1.96	3.21	50.3	51.4	25	38	42
Val	2.71	4.79	69.5	76.7	40	49	63
Trp	1.87 ^1^	nd ^2^	nd ^2^	Nd ^2^	6.6	11	14
Non-essential amino acid							
Asp	4.79	8.07	122.8	129.2			
Glu	6.17	8.53	158.2	136.6			
Ala	3.08	4.84	80.0	77.5			
Arg	2.52	3.92	64.6	62.8			
Gly	2.66	3.72	68.2	59.6			
Pro	2.74	4.10	70.3	65.7			
Ser	1.99	3.16	51.0	50.6			
Kp	4.71	5.36					
Total Nitrogen (%) ^3^	8.28	11.65					
Protein content (%)	39.00	62.44					
Extraction yield (%)	35.8						
Protein yield (%)	59.3						

^1^ value is referenced from Janssen et al. [38]; ^2^ not determined; ^3^ sum of sulphur amino acids (Cys and Met); ^4^ sum of aromatic amino acids (Phe and Try); ^5^ values from Young and Pellet [44].

## Data Availability

Data is contained within the article or supplementary material.

## Supplementary Materials

The following are available online at https://www.mdpi.com/article/10.3390/foods10051048/s1, Figure S1: Zeta potential of LO emulsions (0.2 M phosphate buffer pH 7) versus storage time.

## References

[1] United Nations (2019). World Population Prospects 2019.

[2] Alexandratos N., Bruinsma J. (2012). World Agriculture towards 2030/2050 the 2012 Revision.

[3] Charlton A.J., Dickinson M., Wakefield M.E., Fitches E., Kenis M., Han R., Zhu F., Kone N., Grant M., Devic E. (2015). Exploring the chemical safety of fly larvae as a source of protein for animal feed. J. Insects Food Feed.

[4] Oonincx D.G.A.B., Van Broekhoven S., Van Huis A., Van Loon J.J.A. (2015). Feed conversion, survival and development, and composition of four insect species on diets composed of food by-products. PLoS ONE.

[5] Spranghers T., Ottoboni M., Klootwijk C., Ovyn A., Deboosere S., De Meulenaer B., Michiels J., Eeckhout M., De Clercq P., De Smet S. (2017). Nutritional composition of black soldier fly (Hermetia illucens) prepupae reared on different organic waste substrates. J. Sci. Food Agric..

[6] Van Huis A., Van Itterbeeck J., Klunder H., Mertens E., Halloran A., Muir G., Vantomme P. (2013). Edible Insects: Future Prospects for Food and Feed Security.

[7] Van Krimpen M., Bikker P., Van der Meer I., Van der Peet-Schwering C., Vereijken J. (2013). Cultivation, Processing and Nutritional Aspects for Pigs and Poultry of European Protein Sources as Alternatives for Imported Soybean Products.

[8] Zielińska E., Baraniak B., Karaś M., Rybczyńska K., Jakubczyk A. (2015). Selected species of edible insects as a source of nutrient composition. Food Res. Int..

[9] Amato M. (2017). Insects as Food: A Cross-Cultural Comparison of Consumers’ Intention and Behaviour. Ph.D. Thesis.

[10] DeFoliart G.R. (1999). Insects as food: Why the Western Attitude Is Important. Annu. Rev. Entomol..

[11] Hartmann C., Shi J., Giusto A., Siegrist M. (2015). The psychology of eating insects: A cross-cultural comparison between Germany and China. Food Qual. Prefer..

[12] Tan H.S.G., Fischer A.R.H., Tinchan P., Stieger M., Steenbekkers L.P.A., van Trijp H.C.M. (2015). Insects as food: Exploring cultural exposure and individual experience as determinants of acceptance. Food Qual. Prefer..

[13] Yen A.L. (2009). Edible insects: Traditional knowledge or western phobia?. Entomol. Res..

[14] Balzan S., Fasolato L., Maniero S., Novelli E. (2016). Edible insects and young adults in a north-east Italian city an exploratory study. Br. Food J..

[15] Schösler H., de Boer J., Boersema J.J. (2012). Can we cut out the meat of the dish? Constructing consumer-oriented pathways towards meat substitution. Appetite.

[16] Bußler S., Rumpold B.A., Jander E., Rawel H.M., Schlüter O.K. (2016). Recovery and techno-functionality of flours and proteins from two edible insect species: Meal worm (Tenebrio molitor) and black soldier fly (Hermetia illucens) larvae. Heliyon.

[17] Caligiani A., Marseglia A., Leni G., Baldassarre S., Maistrello L., Dossena A., Sforza S. (2018). Composition of black soldier fly prepupae and systematic approaches for extraction and fractionation of proteins, lipids and chitin. Food Res. Int..

[18] Gould J., Wolf B. (2018). Interfacial and emulsifying properties of mealworm protein at the oil/water interface. Food Hydrocoll..

[19] Hall F.G., Jones O.G., O’Haire M.E., Liceaga A.M. (2017). Functional properties of tropical banded cricket (Gryllodes sigillatus) protein hydrolysates. Food Chem..

[20] Chou T.H., Nugroho D.S., Cheng Y.S., Chang J.Y. (2020). Development and Characterization of Nano-emulsions Based on Oil Extracted from Black Soldier Fly Larvae. Appl. Biochem. Biotechnol..

[21] Park S.I., Kim J.W., Yoe S.M. (2015). Purification and characterization of a novel antibacterial peptide from black soldier fly (Hermetia illucens) larvae. Dev. Comp. Immunol..

[22] Mishyna M., Martinez J.-J.J.I., Chen J., Benjamin O. (2019). Extraction, characterization and functional properties of soluble proteins from edible grasshopper (Schistocerca gregaria) and honey bee (Apis mellifera). Food Res. Int..

[23] Purschke B., Tanzmeister H., Meinlschmidt P., Baumgartner S., Lauter K., Jäger H. (2018). Recovery of soluble proteins from migratory locust (Locusta migratoria) and characterisation of their compositional and techno-functional properties. Food Res. Int..

[24] Zielińska E., Karaś M., Baraniak B. (2018). Comparison of functional properties of edible insects and protein preparations thereof. LWT.

[25] Purschke B., Meinlschmidt P., Horn C., Rieder O., Jäger H. (2018). Improvement of techno-functional properties of edible insect protein from migratory locust by enzymatic hydrolysis. Eur. Food Res. Technol..

[26] Kim H., Setyabrata D., Lee Y.J., Jones O.G., Kim Y.H.B. (2016). Pre-treated mealworm larvae and silkworm pupae as a novel protein ingredient in emulsion sausages. Innov. Food Sci. Emerg. Technol..

[27] Scholliers J., Steen L., Glorieux S., Van de Walle D., Dewettinck K., Fraeye I. (2019). The effect of temperature on structure formation in three insect batters. Food Res. Int..

[28] Wang Y.-S., Shelomi M. (2017). Review of Black Soldier Fly (Hermetia illucens) as Animal Feed and Human Food. Foods.

[29] Ramos-Elorduy J., Manuel J., Moreno P., Prado E.E., Perez M.A., Otero J.L., Guevara O.L. (1997). De Nutritional Value of Edible Insects from the State of Oaxaca, Mexico. J. Food Compos. Anal..

[30] Nazir A., Schroën K., Boom R. (2010). Premix emulsification: A review. J. Memb. Sci..

[31] Kaade W., Güell C., Ballon A., Mellado-Carretero J., De Lamo-Castellví S., Ferrando M. (2020). Dynamic membranes of tunable pore size for lemon oil encapsulation. LWT.

[32] Wang J., Martínez-Hernández A., de Lamo-Castellví S., Romero M.-P., Kaade W., Ferrando M., Güell C. (2020). Low-energy membrane-based processes to concentrate and encapsulate polyphenols from carob pulp. J. Food Eng..

[33] Ladjal-Ettoumi Y., Berton-Carabin C., Chibane M., Schroën K., Ladjal Ettoumi Y., Berton-Carabin C., Chibane M., Schroën K. (2017). Legume Protein Isolates for Stable Acidic Emulsions Prepared by Premix Membrane Emulsification. Food Biophys..

[34] Zhao X., Vázquez-Gutiérrez J.L., Johansson D.P., Landberg R., Langton M. (2016). Yellow mealworm protein for food purposes—Extraction and functional properties. PLoS ONE.

[35] Lourenço S.O., Barbarino E., De-Paula J.C., Pereira L.O.D.S., Lanfer Marquez U.M., Lourenco S.O., Barbarino E., De-Paula J.C., da S. Pereira L.O., Marquez U.M.L. (2002). Amino acid composition, protein content and calculation of nitrogen-to-protein conversion factors for 19 tropical seaweeds. Phycol. Res..

[36] Mellado-Carretero J., García-Gutiérrez N., Ferrando M., Güell C., García-Gonzalo D., De Lamo-Castellví S. (2020). Rapid discrimination and classification of edible insect powders using ATR-FTIR spectroscopy combined with multivariate analysis. J. Insects Food Feed.

[37] Kaade W., Ferrando M., Khanmohammed A., Torras C., De Lamo-Castellví S., Güell C. (2019). Low-energy high-throughput emulsification with nickel micro-sieves for essential oils encapsulation. J. Food Eng..

[38] Janssen R.H., Vincken J.-P.P., Van Den Broek L.A.M.M., Fogliano V., Lakemond C.M.M.M. (2017). Nitrogen-to-Protein Conversion Factors for Three Edible Insects: Tenebrio molitor, Alphitobius diaperinus, and Hermetia illucens. J. Agric. Food Chem..

[39] Oshimura E., Sakamoto K. (2017). Amino acids, peptides, and proteins. Cosmet. Sci. Technol. Theor. Princ. Appl..

[40] Burns A., Olszowy P., Ciborowski P. (2016). Biomolecules. Proteomic Profiling and Analytical Chemistry: The Crossroads.

[41] Azagoh C., Ducept F., Garcia R., Rakotozafy L., Cuvelier M.-E., Keller S., Lewandowski R., Mezdour S. (2016). Extraction and physicochemical characterization of Tenebrio molitor proteins. Food Res. Int..

[42] Yi L., Lakemond C.M.M., Sagis L.M.C., Eisner-Schadler V., Van Huis A., Boekel M.A.J.S.V. (2013). Extraction and characterisation of protein fractions from five insect species. Food Chem..

[43] Laroche M., Perreault V., Marciniak A., Gravel A., Chamberland J., Doyen A. (2019). Comparison of conventional and sustainable lipid extraction methods for the production of oil and protein isolate from edible insect meal. Foods.

[44] Young V.R., Pellett P.L. (1991). Protein evaluation, amino acid scoring and the Food and Drug Administration’s proposed food labeling regulations. J. Nutr..

[45] Chatsuwan N., Nalinanon S., Puechkamut Y., Lamsal B.P., Pinsirodom P. (2018). Characteristics, Functional Properties, and Antioxidant Activities of Water-Soluble Proteins Extracted from Grasshoppers, Patanga succincta and Chondracris roseapbrunner. J. Chem..

[46] Socrates G. (2004). Infrared and Raman Characteristic Group Frequencies Contents.

[47] Haque M.A., Timilsena Y.P., Adhikari B. (2016). Food Proteins, Structure, and Function. Reference Module in Food Science.

[48] Son Y.J., Lee J.C., Hwang I.K., Nho C.W., Kim S.H. (2019). Physicochemical properties of mealworm (Tenebrio molitor) powders manufactured by different industrial processes. LWT.

[49] Chandra S. (2013). Assessment of functional properties of different flours. Afr. J. Agric. Res..

[50] McClements D.J., Gumus C.E. (2016). Natural emulsifiers—Biosurfactants, phospholipids, biopolymers, and colloidal particles: Molecular and physicochemical basis of functional performance. Adv. Colloid Interface Sci..

[51] Onwulata C.I. (2013). Microencapsulation and functional bioactive foods. J. Food Process. Preserv..

[52] Rao J., McClements D.J. (2012). Lemon oil solubilization in mixed surfactant solutions: Rationalizing microemulsion & nanoemulsion formation. Food Hydrocoll..

[53] Burt S. (2004). Essential oils: Their antibacterial properties and potential applications in foods—A review. Int. J. Food Microbiol..

[54] Nazir A., Boom R.M., Schroën K. (2013). Droplet break-up mechanism in premix emulsification using packed beds. Chem. Eng. Sci..

[55] Sahin S., Sawalha H., Schroën K. (2014). High throughput production of double emulsions using packed bed premix emulsification. Food Res. Int..

[56] Rao J., McClements D.J. (2012). Impact of lemon oil composition on formation and stability of model food and beverage emulsions. Food Chem..

[57] Treesuwan W., Neves M.A., Uemura K., Nakajima M., Kobayashi I. (2017). Preparation characteristics of monodisperse oil-in-water emulsions by microchannel emulsification using different essential oils. LWT Food Sci. Technol..

[58] González-Mas M.C., Rambla J.L., López-Gresa M.P., Amparo Blázquez M., Granell A. (2019). Volatile compounds in citrus essential oils: A comprehensive review. Front. Plant. Sci..

[59] Cheong M.W., Chong Z.S., Liu S.Q., Zhou W., Curran P., Yu B. (2012). Characterisation of calamansi (Citrus microcarpa). Part I: Volatiles, aromatic profiles and phenolic acids in the peel. Food Chem..

[60] Diamante L.M., Lan T. (2014). Absolute Viscosities of Vegetable Oils at Different Temperatures and Shear Rate Range of 64.5 to 4835 s-1. J. Food Process..

[61] Trentin A., Ferrando M., López F., Güell C. (2009). Premix membrane O/W emulsification: Effect of fouling when using BSA as emulsifier. Desalination.

[62] Surh J., Jeong Y.G., Vladisavljević G.T. (2008). On the preparation of lecithin-stabilized oil-in-water emulsions by multi-stage premix membrane emulsification. J. Food Eng..

[63] Sawalha H., Sahin S., Schroën K. (2016). Preparation of polylactide microcapsules at a high throughput with a packed-bed premix emulsification system. J. Appl. Polym. Sci..

[64] Zecca E. (2017). Investigating the Role of Surface Hydrophobicity in Protein Aggregation. Ph.D. Thesis.

