# Supplementary material for: Black Soldier Fly (Hermetia illucens) Protein Concentrates as a Sustainable Source to Stabilize O/W Emulsions Produced by a Low-Energy High-Throughput Emulsification Technology
Source: Foods. 2021 May 11;10(5):1048. doi: 10.3390/foods10051048 (PMC8151181; doi:10.3390/foods10051048)
Supplement: Supplementary file 1 [file foods-10-01048-s001.zip › foods-1196238-supplementary.pdf]

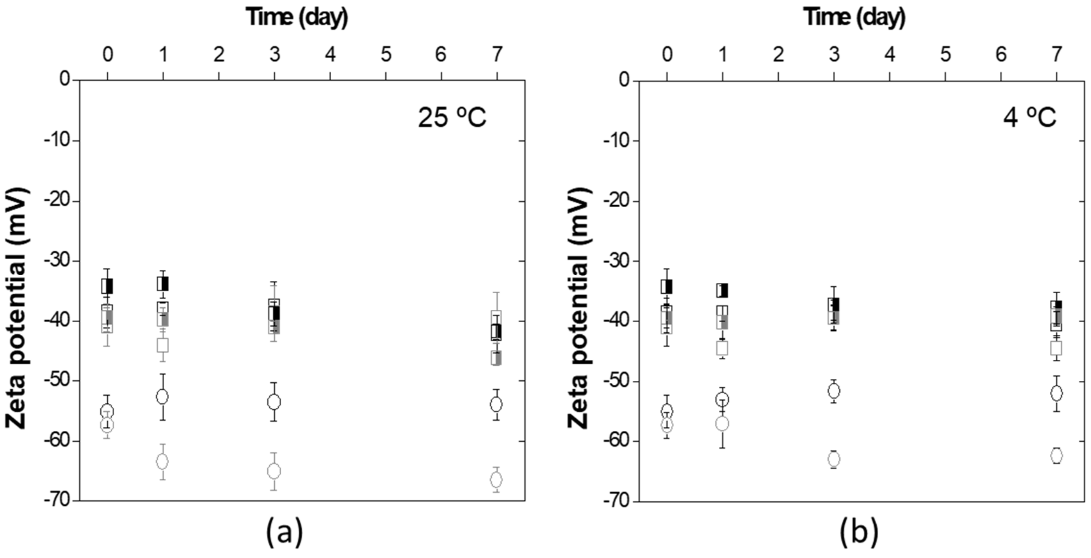

Figure S1. Zeta potential of LO emulsions (0.2 M phosphate buffer pH 7) versus storage time: (a) at room temperature (25 °C); (b) in fridge (4 °C). (○20%LO-1% WPI;□20%LO-1% BSFPC; ◨ 20%LO-2% BSFPC;○40%LO-1% WPI; □ 40%LO-1% BSFPC; ◨ 40%LO-2%BSFPC)
